# Hepatic acute phase response protects the brain from focal inflammation during postnatal window of susceptibility

**DOI:** 10.1016/j.bbi.2018.01.008

**Published:** 2018-03

**Authors:** Inês Sá-Pereira, Jay Roodselaar, Yvonne Couch, Marcia Consentino Kronka Sosthenes, Matthew C. Evans, Daniel C. Anthony, Helen B. Stolp

**Affiliations:** a Department of Pharmacology, University of Oxford, United Kingdom; b Acute Stroke Programme, Radcliffe Department of Medicine, University of Oxford, United Kingdom; c Universidade Federal do Pará, Laboratório de Investigações em Neurodegeneração e Infecção, ICB/HUJBB, Belém, Brazil; d Centre for the Developing Brain, Division of Imaging Sciences and Biomedical Engineering, St Thomas’ Hospital, King’s College London, United Kingdom; e Royal Veterinary College, London, United Kingdom

**Keywords:** APR, acute phase response, CNS, central nervous system, CXCL, C-X-C motif ligand, IL, interleukin, ICAM, intercellular adhesion molecule, P, postnatal day, LPS, lipopolysaccharide, PBS, phosphate-buffered saline, rrIL-1β, rat recombinant IL-1β, RT-qPCR, Real-Time polymerase chain reaction, SEM, standard error of the mean, TNF, tumour necrosis factor, Blood-brain barrier, Acute phase response, Neutrophil recruitment, Brain development, Neuroinflammation

## Abstract

Perinatal inflammation is known to contribute to neurodevelopmental diseases. Animal models of perinatal inflammation have revealed that the inflammatory response within the brain is age dependent, but the regulators of this variation remain unclear. In the adult, the peripheral acute phase response (APR) is known to be pivotal in the downstream recruitment of leukocytes to the injured brain. The relationship between perinatal brain injury and the APR has not been established. Here, we generated focal inflammation in the brain using interleukin (IL)-1β at postnatal day (P)7, P14, P21 and P56 and studied both the central nervous system (CNS) and hepatic inflammatory responses at 4 h. We found that there is a significant window of susceptibility in mice at P14, when compared to mice at P7, P21 and P56. This was reflected in increased neutrophil recruitment to the CNS, as well as an increase in blood–brain barrier permeability. To investigate phenomena underlying this window of susceptibility, we performed a dose response of IL-1β. Whilst induction of endogenous IL-1β or intercellular adhesion molecule (ICAM)-1 in the brain and induction of a hepatic APR were dose dependent, the recruitment of neutrophils and associated blood–brain barrier breakdown was inversely proportional. Furthermore, in contrast to adult animals, an additional peripheral challenge (intravenous IL-1β) reduced the degree of CNS inflammation, rather than exacerbating it. Together these results suggest a unique window of susceptibility to CNS injury, meaning that suppressing systemic inflammation after brain injury may exacerbate the damage caused, in an age-dependent manner.

## Highlights

• Postnatal mouse brain is most susceptible to an acute inflammatory challenge at P14.
• CNS neutrophil number inversely correlates with systemic, not central, inflammation.
• Systemic activation of the acute phase response suppresses CNS inflammation at P14.
• Anti-inflammatory therapy after CNS injury may be sensitive to developmental stage.

## 1 Introduction

Neuroinflammation is implicated in the aetiology of neurodevelopmental disorders, such as autism, schizophrenia (Bayer et al., 1999, Bloomfield et al., 2016, Estes and McAllister, 2015, Fillman et al., 2013, Trépanier et al., 2016, Vargas et al., 2005) and cerebral palsy (Kadhim et al., 2003, Kadhim et al., 2001, Mallard et al., 2014). The impact of inflammation on development appears to be highly dependent on the timing of the challenge; for instance, autism and schizophrenia have been associated with infection during the first and second trimester, while vulnerability to cerebral palsy was highest in the last trimester and early postnatal period (Atladóttir et al., 2010, Brown et al., 2004, Dubowitz et al., 1985, Mednick et al., 1988).

Inflammatory challenges in rodents at different time points during development have revealed that there are striking differences in the nature of the cellular and molecular responses (Lawson and Perry, 1995, Meyer et al., 2006, Straley et al., 2017). For example, a window of susceptibility exists in rats at three-weeks postpartum, when the generation of a focal inflammatory lesion in the brain results in increased leukocyte recruitment and blood–brain barrier breakdown, which is not observed either before or after this window (Anthony et al., 1997). Peripheral immune activation by the intraperitoneal injection of lipopolysaccharide (LPS) has also been shown to transiently increased blood–brain barrier permeability in the periventricular white matter tracts during the first postnatal week in rats (Stolp et al., 2005) and an intraperitoneal injection of LPS followed by hypoxia–ischemia was shown to increase blood–brain barrier permeability at postnatal day (P)12 but not at P1 in Lewis rats (Brochu et al., 2011). Both studies from Anthony et al., 1997, Brochu et al., 2011 have correlated blood–brain barrier permeability with the recruitment of neutrophils to the brain. These variations in the inflammatory response during development may alter cortical development and have a lasting impact on behaviour (Stolp et al., 2011a, Stolp et al., 2011b).

In adults, focal cerebral inflammation (Campbell et al., 2005, Campbell et al., 2003, Wilcockson et al., 2002), traumatic brain injury (Villapol et al., 2015) and cerebral ischemia (Chapman et al., 2009) in rodents have been shown to induce the hepatic acute phase response (APR) characterized by the expression of cytokines and chemokines, other acute phase proteins such as serum amyloid A, and the recruitment of neutrophils and macrophages. Campbell et al. (2003) observed that an intracerebral injection of interleukin (IL)-1β induced the production and release of C-X-C motif ligand (CXCL)-1 by the liver and the recruitment of neutrophils to the liver, blood and brain in a dose-dependent manner. The hepatic APR functions to eliminate the inflammatory stimuli, attenuate local inflammation, and promote tissue repair and regeneration (Anthony et al., 2012, Anthony and Couch, 2014). However, by recruiting and priming leukocytes to the site of injury in the central nervous system (CNS), the APR may also contribute to further damage in the brain. Systemic inflammation has been shown to exacerbate focal neuroinflammatory injury in the adult. A number of rodent and human studies have demonstrated a positive correlation between the magnitude of the APR and brain injury (Acalovschi et al., 2003, Campbell et al., 2003, Di Napoli et al., 2005, Smith et al., 2006, Smith et al., 2004, Vila et al., 2000, Villapol, 2016).

In the perinatal period, an altered hepatic APR has also been reported following hypoxic-ischemic encephalopathy in rats, which was characterized by upregulation of CXCL-1 and downregulation of tumour necrosis factor (TNF), IL-1β and CCL-2 (Bonestroo et al., 2013). However, the nature of this response, and how it affects the evolution of the central injury, is still unclear. It is likely that, as in adult, the hepatic APR may impact the magnitude of the CNS immune response and subsequent secondary injury.

In this study, we use a well-established model of focal intracerebral inflammation to study the role of the hepatic APR in modulating the brain response to acute inflammation during postnatal development. The intrastriatal injection of IL-1β generates a reproducible focal inflammatory lesion which is accompanied by de novo production of cytokines, the activation of microglia and local endothelial cells in the absence of acute neuronal cell death (Campbell et al., 2007a, Campbell et al., 2007b, Docagne et al., 2005, van Kasteren et al., 2009). The local cellular response to the inflammatory challenge is conspicuous from 4 h to 7 days post injection (Docagne et al., 2005). Here we show that susceptibility of the brain to focal inflammation varies with age, and that contrary to expectations, the CNS response is inversely proportional to the APR during the window of susceptibility.

## 2 Materials and methods

### 2.1 Animals

C57Bl/6 mice were used at postnatal day (P)7, P14, P21 and P56. These ages were selected based on a previous publication in rats (Anthony et al., 1997), and on preliminary data from our own lab in mice, showing a mid-to-late postnatal window of susceptibility to an inflammatory challenge that is likely to be related to the increased susceptibility of children to CNS infections and injury (Catroppa et al., 2008, Duval et al., 2008, Semple et al., 2013). Mice were obtained from Harlan, UK, and acclimatised for at least 3 days to the local environment prior to experimentation. Animals were housed in specific pathogen-free facilities under a standard light/dark cycle with food and water ad libitum. All animal procedures were conducted in accordance with the U.K. Animals (Scientific Procedures) Act, 1986 and associated guidelines, EU Directive 2010/63/EU for animal experiments (PPL: 30/3076). Both male and female pups were used in these experiments; no effect of sex was identified in any response measured. Litters were split between experimental groups to limit any effect of maternal care on the experiments. The number of animals used in each group studied can be found in figure legends.

### 2.2 Administration of IL-1β

Animals were anaesthetised with 2–3% isoflurane in oxygen. Stereotaxic surgery was performed under an operating microscope, as previously described (Couch et al., 2014). The top of the head was shaved and positioned in a stereotaxic frame. The skull was exposed and a small hole made with dental drill burr (Bregma: Anterior/Posterior + 0.5 mm; Medial/Lateral + 1 mm (P7, P14), 1.5 mm (P21) or 1.8 mm (P56); Dorsal/Ventral − 1.7 mm (P7) or 2 mm (P14, P21, P56). Rat recombinant IL-1β (rrIL-1β, 501-RL/CF; 2 ng/µl, 20 ng/µl or 200 ng/µl; R&D Systems, UK) or vehicle (0.9% saline) were injected into the left striatum with finely-drawn glass microcapillary needle (0.5 µl over a period of 5 min), and the wound was closed using surgical clips. All coordinates were defined using dye injections in preliminary experiments. In a cohort of mice at P14, saline or 100 ng of rrIL-1β (50 µl) was injected into the tail vein immediately after the intracerebral injection outlined above. This dose of IL-1β was chosen based on previous results showing that 100 ng of IL-1β induces the hepatic expression of factor nuclear kappa B (NFkB), which regulates neutrophil recruitment to the injured brain, at a similar level to the focal intracerebral injection of IL-1β (Campbell et al., 2008). Animals recovered in a heated chamber before being returned to their cage.

### 2.3 Tissue preparation

Two or four hours following intracerebral or intravenous injection of rrIL-1β or saline, mice were anaesthetized with pentobarbital (20% w/v pentobarbital sodium, i.p.; J M Loveridge Ltd, UK) and then transcardially perfused with cold 0.9% saline with heparin (Sigma, UK). Liver and striatum were frozen on dry ice for mRNA extraction. The striatum was dissected into ipsilateral (injected) and contralateral side from saline and IL-1β injected animals, and the contralateral (right) side from naïve animals. Alternately, mice were transcardially perfused fixed with 4% paraformaldehyde (in 0.1 M phosphate buffer, pH 7.4) after 0.9% saline with heparin (Sigma, UK). Brain and liver were collected and immersed into 4% paraformaldehyde at 4 °C for 24 h, before cryoprotection in 30% sucrose (4 °C) and subsequently embedded in O.C.T. compound (CellPath, UK). Ten-micrometer-thick sections were cut on a cryostat Leica CM1850 (Leica Microsystems, UK) and mounted on gelatin-coated slides in a serial manner. Brain sections were collected for the full extent of the injection site and lesion, from approximately Bregma + 1.10 to − 0.10, based on The Mouse Brain in Stereotaxic Coordinates (Paxinos and Franklin).

### 2.4 Immunohistochemistry and light-microscopy

Frozen sections were dried (37 °C, 15 min), quenched for endogenous peroxidase activity (0.3% hydrogen peroxide in methanol; Sigma, UK) and blocked for non-specific Fc-dependent binding in 10% serum in phosphate-buffered saline (PBS). Endogenous biotin in the liver was blocked using an avidin–biotin blocking kit (Vector Laboratories, UK). Primary antibodies (laminin, 1:500, Abcam ab7463; anti-neutrophil serum HB199, 1:1000, made in-house, in rabbits) were incubated at 4 °C overnight. Sections were incubated in biotinylated secondary antibody for 2 h at room temperature (Vector Laboratories, UK), followed by an avidin–biotin-peroxidase solution (Vectastain Elite ABC, Vector Laboratories, UK) for 1 h at room temperature and the reaction product detected with diaminobenzidine hydrochloride (DAB, 0.5 µg/ml, Sigma, UK) in 0.1 M phosphate buffer with 0.05% hydrogen peroxide, with PBS washes (3x5min) between each step. Slides were counterstained with cresyl violet (Acros Organics, Fisher Scientific, UK), dehydrated and cleared before permanent mounting with DPX medium. Blood-brain barrier permeability was measured by determining the extent of endogenous IgG in the brain. Tissue was incubated with a biotinylated antibody against mouse IgG (Vector Laboratories, UK). Double immunohistochemistry for neutrophils and laminin was performed by two sequential immunohistochemistry processes using two different peroxidase substracts: DAB (brown) and VECTOR VIP (purple; Vector Laboratories, UK), respectively. These were separated by an avidin/biotin blocking (Avidin/Biotin blocking kit, Vector Laboratories, UK). A citrate buffer (10 mM Citric Acid, 0.05% Tween 20, pH 6.0) antigen retrieval step was added to the laminin staining protocol.

Cell counts were performed using a Leitz Laborlux S microscope (Leica Biosystems, GE) under 40 × magnification. Neutrophils were randomly counted throughout the liver using a 10 × 10 eyepiece grid that equated to an area of 0.063 mm^2^. The total number of neutrophils in the parenchyma and/or vessels (either in the lumen or attached to the vessel wall) in the injection site were counted in the whole ipsilateral striatum of at least two coronal sections for each brain, approximately 120 µm apart. The striatum was traced under a Camera Lucida (4 × magnification) attached to the microscope. All tracings were scanned and the area was measured in ImageJ. Calibration was performed with a 1 mm scale micrometer slide in order to obtain the final number of cells per mm^2^ of striatum.

To schematically represent neutrophil recruitment patterns in the brain, a spatial map of neutrophil location was drawn using a Camera Lucida attached to the light microscope from a coronal section at Bregma + 0.5 mm through the injection site. Spatial maps were superimposed onto coronal figure 27 from The Mouse Brain in Stereotaxic Coordinates (Paxinos and Franklin) using Adobe Photoshop.

### 2.5 Real-Time polymerase chain reaction (RT-qPCR)

mRNA was isolated using RNeasy mini kit (Qiagen, UK) following manufacturer’s guidelines. The quantity and quality of mRNA were assessed using a NanoDrop 1000 Spectrophotometer (Thermo Scientific, UK). mRNA was deemed to have sufficient purity when the absorbance ratio at 260:280 was ∼ 2.0 and 260:230 ratio was 1.8–2.2. mRNA was converted to cDNA using nanoScript2™ reverse transcript kit (Primerdesign, UK). Real-Time polymerase chain reaction (RT-qPCR) was performed using a LightCycler 480 (Roche Diagnostics, UK) and SBYR green mastermix (Primerdesign, UK). Primers for intercellular adhesion molecule-1 (ICAM-1) were obtained from Invitrogen and, primers for TNF were obtained from Roche Life Science. The rest of the primers were obtained as custom designed real-time PCR assays from Primerdesign (IL-1β: GCTATGGCAACTGTTCCTGAA, R: ACAGCCCAGGTCAAAGGTT; IL-6: AATTCCAGAAACCGCTATGAAGT and ATCCTCTGTGAAGTCTCCTCTC; TNF: TGCCTATGTCTCAGCCTCTTC and GAGGCCATTTGGGAACTTCT; CXCL-2: CAGAAGTCATAGCCACTCTCAAG and AGCCTTGCCTTTGTTCAGTATC; CXCL-5: AAAGATTTCTGAGGACTCTGACC and TTTTCTCATCAAAGCAGGGAGT; ICAM-1: CCCACGCTACCTCTGCTC and GATGGATACCTGAGCATCACC). House-keeping genes were selected by a GeNorm analysis using a representative set of brain and liver samples (geNormPLUS 12 gene kit, Primerdesign, UK). GeNorm allows the selection of one or more candidate reference genes that are not affected by the experimental conditions and constitutively expressed in all the samples. Zinc finger protein 91 (Zpf91) and cell division cycle 40 homolog (yeast, Cdc40) were used as housekeeping genes for brain samples and, casein kinase 2, alpha prime polypeptide (Csnk2a2) and PAK1 interacting protein 1 (Pak1ip1) for liver samples. A relative quantification was performed using ΔΔCt analysis. The results were represented as fold change from naïve (or other relevant controls, as stated in the results) expression levels.

### 2.6 Statistics

Data are presented as mean ± standard error of the mean (SEM) and considered statistically significant with a p < .05. Data were analysed using one-way ANOVA with Tukey’s multiple comparison test or two-way ANOVA with Bonferroni–Dunn post hoc test for multiple groups where appropriate.

## 3 Results

### 3.1 Neutrophil recruitment and increases in blood–brain barrier permeability following intracerebral IL-1β are age-dependent

**Fig. 1:**
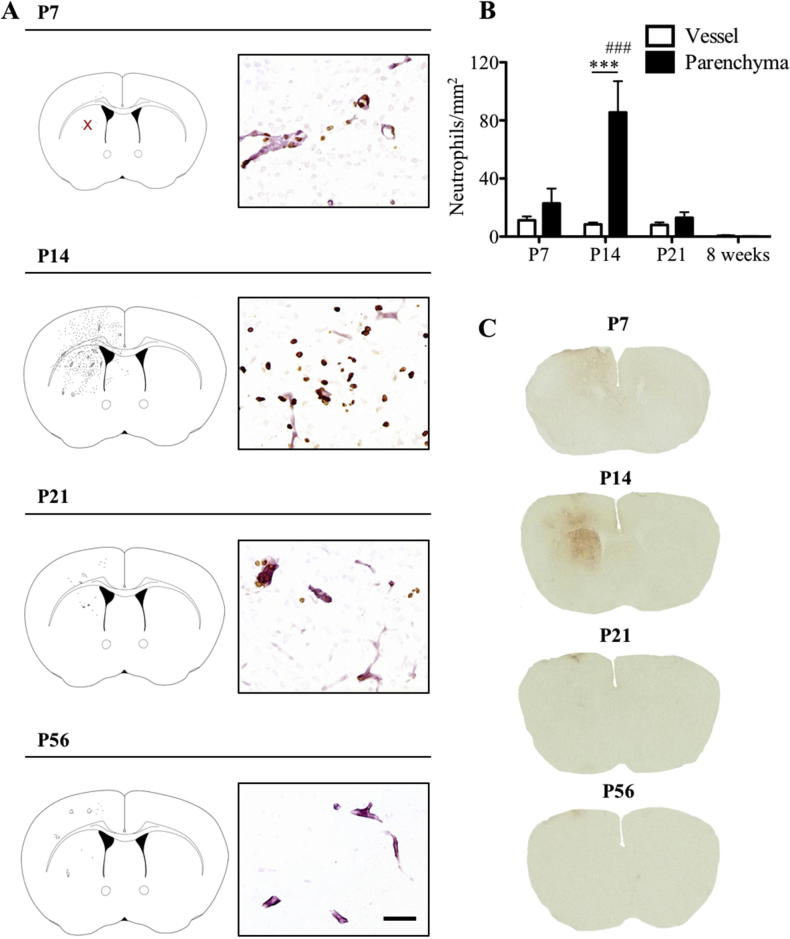
Recruitment of neutrophils to brain parenchyma and permeability of the blood–brain barrier to endogenous IgG 4 h after focal inflammation during postnatal period. Neutrophils were recruited to the site of the intracerebral IL-1β (1ng) injection as shown in representative spatial maps of neutrophil distribution in the brain (A). Recruitment of neutrophils 4 h after the injection was highest at P14 (A, B) and they were found to enter the brain parenchyma as well as filling the vessel lumen. Photomicrographs in (A) show neutrophils (brown; anti-neutrophil serum) and vessels (pink; laminin) within the striatal injection site (marked by red ‘x’ in the P7 spatial map). Intracerebral injection of 1 ng IL-1β and subsequent neutrophil recruitment was associated with increased blood–brain barrier permeability to endogenous IgG at P14, but not at P7, P21 and P56 as shown in the photomicrographs in (C). Scale bar = 50 µm. ^***^p < .001. ^###^p < .001 vs. all parenchyma values. P7: n = 4, P14: n = 5, P21: n = 4 and P56: n = 3. (For interpretation of the references to colour in this figure legend, the reader is referred to the web version of this article.)

Focal inflammation in the brain was induced by injecting 1 ng IL-1β into the left striatum of C57Bl/6 mice at P7, P14, P21 and P58. Naïve and saline injected brains were used as controls. Neutrophil recruitment to the striatum was assessed 4 h after intracerebral injection (Fig. 1). No neutrophils were observed in the brain (blood vessels or parenchyma) of naïve or saline injected mice. Following a 1 ng IL-1β injection into the brain, there was significantly (p < .001) enhanced neutrophil recruitment at P14 (85 ± 21 cells/mm^2^), compared to P7 (23 ± 10 cells/mm^2^), P21 (13 ± 4 cells/mm^2^) or P56 (0.1 ± 0.1 cells/mm^2^, Fig. 1A, B and Supplementary Fig. 1). However, while there was a marked increase at P14 compared to the other early perinatal time points, the recruitment at all perinatal ages was markedly increased compared to the recruitment observed in the adult animals. In view of the age-dependent changes in neutrophil recruitment to the brain, permeability of the blood–brain barrier to endogenous IgG was also assessed (Fig. 1C). In control brains, endogenous IgG could not be detected macroscopically. However, injection of 1 ng IL-1β increased blood–brain barrier permeability in the ipsilateral striatum at P14, but not at other ages evaluated, or in the contralateral striatum. These results indicate that injection of 1 ng IL-1β induced an acute window of susceptibility at P14 in mice, which was characterized by excessive recruitment of neutrophils to the injected striatum and increased blood–brain barrier permeability to endogenous IgG when compared to earlier and later stages of development.

### 3.2 The window of susceptibility at P14 disappears with increasing doses of intracerebral IL-1β

**Fig. 2:**
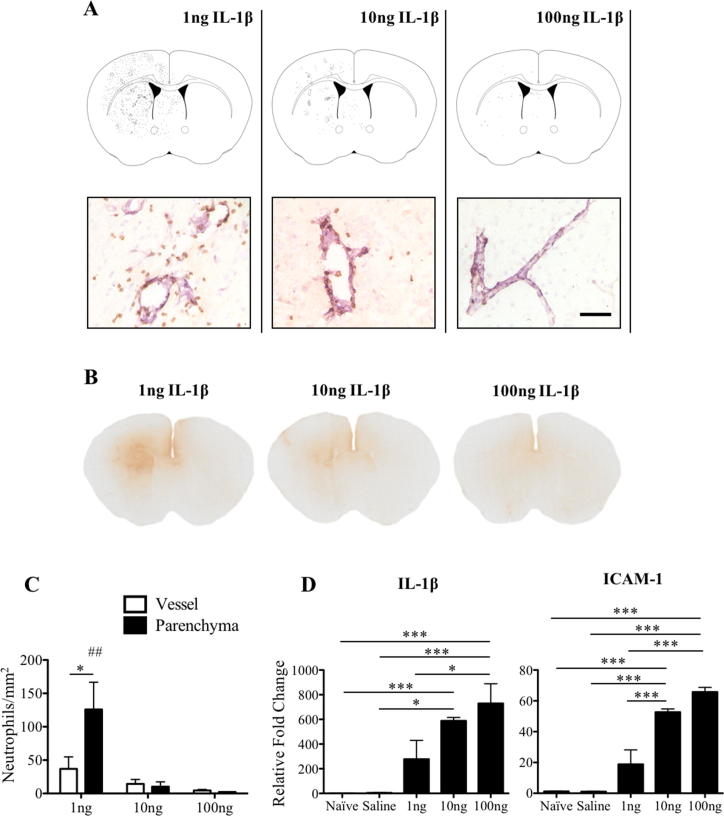
Effects of intracerebral injection of increasing doses of IL-1β on blood–brain barrier permeability, recruitment of neutrophils and expression of IL-1β and ICAM-1 at P14. At 4 h an inverse relationship was found to be present between the intracerebral dose of IL-1β and subsequent neutrophil recruitment and blood–brain barrier breakdown at P14 (A–C). (A) Representative spatial maps of neutrophil distribution in the brain and micrographs of tissue stained for neutrophils (brown; anti-neutrophil serum) and vessels (pink; laminin), showing extensive neutrophil recruitment with 1 ng IL-1β, which reduced as the dose of IL-1β was increased. (B) Immunohistochemistry for IgG shows entry of the endogenous protein into the brain tissue after the intracerebral injection of IL-1β at all doses, with the most staining observed following the 1 ng dose of IL-1β. (C) Neutrophils were found in significantly larger numbers in both the vessel lumen and brain parenchyma in the 1 ng IL-1β group compared to higher doses. (D) IL-1β and ICAM-1 mRNA expression levels were measured in the contralateral striatum of naïve mice and ipsilateral striatum of mice that received intracerebral injection of saline or 1 ng, 10 ng or 100 ng IL-1β at P14. Data are represented as relative fold change from contralateral expression in naïve mice, and show a dose dependent increase in brain gene expression with intracerebral IL-1β injection. Scale bar = 50 µm. ^*^p < .05 and p < .001. ^##^p < .01 vs. all parenchyma values in C. n = 3 for immunohistochemistry analysis; n = 4 for RT-qPCR. (For interpretation of the references to colour in this figure legend, the reader is referred to the web version of this article.)

The relationship between the dose of IL-1β and the brain inflammatory response were investigated at P14. Neutrophil recruitment, blood–brain barrier permeability and gene expression of IL-1β and ICAM-1 were examined in the brain 4 h after intracerebral injection of 1 ng, 10 ng or 100 ng IL-1β and compared to naïve and saline injected mice. Surprisingly, the number of neutrophils recruited to the ipsilateral striatum decreased with increasing dose of IL-1β (Fig. 2A and C). In brains injected with 1 ng IL-1β, 37 ± 18 cells/mm^2^ were observed in vessels and 126 ± 41 cells/mm^2^ were found in the parenchyma (p < .05), indicating that there is no parenchymal barrier to neutrophil recruitment at this age (Fig. 2C). When 10 ng (14 ± 7 cells/mm^2^ in vessels and 10 ± 7 cells/mm^2^ in the parenchyma) or 100 ng IL-1β (5 ± 1/mm^2^ in vessels and 2 ± 0.2/mm^2^ in the parenchyma) was injected in the striatum, not only were there fewer neutrophils present in the brain, but a smaller percentage was able to cross the blood–brain barrier. The response to 10 ng or 100 ng IL-1β was significantly different to the 1 ng dose (p < .01) (Fig. 2C). This result also correlated with the appearance of endogenous IgG in the brain. After 4 h, IgG positive tissue was found in the ipsilateral striatum after 1 ng IL-1β injection, consistent with the data in Fig. 1. Compared to the 1 ng dose, IgG extravasation was decreased when 10 ng IL-1β was injected in the brain, and almost no IgG staining was visible after the 100 ng IL-1β injection (Fig. 2B). This unexpected dose response was further investigated at the other stages of development. We found that, following intracerebral injection of 100 ng IL-1β, there was a statistically significant difference in the parenchymal neutrophil recruitment between the two IL-1β doses administered. At P7, P14 and P21 there was a dose dependent decrease in recruitment at all of these ages. However, at P56 the higher dose of IL-1β resulted in a small, but statistically significant increase in neutrophil recruitment (p < .01) (Supplementary Fig. 2A). No increases in blood–brain barrier permeability to endogenous IgG were observed at any age after intracerebral injection of 100 ng IL-1β (not shown). To test if the absence of the window of susceptibility at 4 h was the result of a change in the kinetics of the response (i.e. earlier neutrophil influx), the recruitment of neutrophils to the brain was also examined 2 h after intracerebral injection of 100 ng IL-1β at P7, P14 and P21. Few neutrophils were found in the brain, with no statistical significance between both time points (2 and 4 h) and the responses in P7, P14 and P21 animals. The lack of neutrophils at 2 h shows that there is no window of susceptibility when compared to 1 ng IL-1β dose at 4 h and suggests that high dose of IL-1β does not change the kinetics of the response (Supplementary Fig. 2B). Therefore, all further experiments were performed at 4 h.

The molecular response to IL-1β-induced inflammation in the brain at P14 was assessed by measuring mRNA expression for IL-1β and for ICAM-1 in the ipsilateral striatum at 4 h (Fig. 2D). In adults, microinjection of IL-1β protein is known to induce further expression of endogenous IL-1β (Blond et al., 2002). Injection of vehicle did not generate an elevation in either transcript (4.57 ± 2.03 for IL-1β and 1.11 ± 0.12 for ICAM-1), compared to naïve P14 brain (1.08 ± 0.06 for IL-1β and 1.24 ± 0.15 for ICAM-1). Expression levels of IL-1β and ICAM-1 increased after stereotaxic microinjection of 1 ng IL-1β (IL-1β: 278.30 ± 151.10; ICAM-1: 18.80 ± 9.36), 10 ng IL-1β (IL-1β: 588.70 ± 27.42; ICAM-1: 52.67 ± 2.10), or 100 ng (IL-1β: 729.80 ± 159.10; ICAM-1: 65.80 ± 2.99) into the striatum. However, the increased expression was only statistically significant with the 10 ng (IL-1β: p < .001 vs. naïve, p < .05 vs. saline; ICAM-1: p < .001 vs. naïve, saline and 1 ng) and 100 ng (IL-1β: p < .001 vs. naïve and saline, p < .05 vs. 1 ng; ICAM-1: p < .001 vs. naïve, saline and 1 ng) doses. Thus, the molecular response is dissociated from the cellular recruitment profile in the perinatal brain.

### 3.3 Intracranial IL-1β induces a hepatic acute phase response at P14 in a dose-dependent manner

**Fig. 3:**
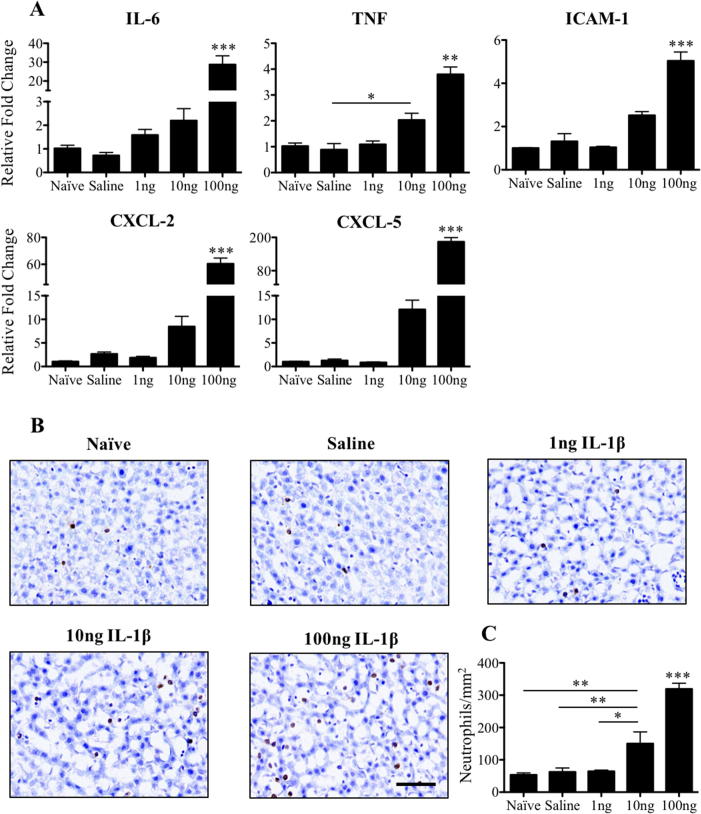
Expression of inflammatory mediators and neutrophil recruitment in the liver 4 h after intracerebral injection of 1 ng, 10 ng or 100 ng IL-1β at P14. IL-6, TNF, ICAM-1, CXCL-2 and CXCL-5 mRNA expression in the liver significantly increases following intracerebral injection of 100 ng of IL-1β, but not after the 1 ng dose (A). Data are represented as relative fold change from expression levels of naïve mice P14. Neutrophil immunohistochemistry, counterstained with cresyl violet, in the liver shows significant increases in the number of neutrophils per mm^2^ in tissue following the intracerebral injection of 10 ng and 100 ng IL-1β at P14. Scale bar = 50 µm. ^*^p < .05, ^**^p < .01 and ^***^p < .001. Naïve, 1 ng, 10 ng and 100 ng: n = 4, and saline: n = 8 for RT-qPCR; naïve: n = 7, saline: n = 6, 1 ng, 10 ng and 100 ng: n = 3 for immunohistochemistry analysis. (For interpretation of the references to colour in this figure legend, the reader is referred to the web version of this article.)

Next, to determine whether the magnitude of the APR in the liver might account for the atypical inflammatory response at P14, the hepatic mRNA expression of the acute phase proteins IL-6, TNF, ICAM-1, CXCL-2 and CXCL-5 was evaluated at 4 h. Intracerebral injection of 1 ng IL-1β into the striatum of P14 animals did not elicit an increase in the hepatic expression of any of the transcripts examined. Injection of 10 ng IL-1β caused a 10-fold increase in the expression of CXCL-2 and CXCL-5, however only TNF expression was statistically different over baseline (saline: 0.88 ± 0.24 and 10 ng: 2.03 ± 0.26, p < .05) following correction for multiple comparisons. In contrast, the 100 ng IL-1β intracerebral injection significantly increased the expression of all transcripts (p < .001) (Fig. 3A). Furthermore, the number of neutrophils found in the liver increased with increasing doses of intracerebral IL-1β (Fig. 3B and C). The number of neutrophils was unchanged in saline (62 ± 13 cells/mm^2^) animals or 1 ng IL-1β injected animals (64 ± 4 cells/mm^2^) compared to naïve (54 ± 6 cells/mm^2^) animals. However, intracerebral injection of either 10 ng IL-1β (150 ± 36 cells/mm^2^, p < .01 vs. naïve and saline, p < .05 vs. 1 ng, p < .001 vs. 100 ng) or 100 ng IL-1β injection (319 ± 18/mm^2^, p < .001 vs. all groups) resulted in a significant increase in the number of hepatic neutrophils, a known feature of the hepatic response to brain injury (Fig. 3C). The hepatic APR to 100 ng IL-1β was not restricted to P14; similar increases in the expression of acute phase proteins transcripts were observed at P7, P21 and P56 for IL-6, TNF, ICAM-1, CXCL-2 and CXCL-5, and was also associated with the recruitment of neutrophils (Supplementary Figs. 3 and 4). However, the magnitude of increase in the expression of the inflammatory mediators and the number of neutrophils present in the liver 4 h after intracerebral injection of 100 ng IL-1β was greatest in the youngest animals studied and decreased with age (statistically significant following two-way ANOVA analysis with Bonferroni–Dunn post hoc test with age and treatment as variables; Supplementary Table 1). There was also greater basal production of the acute phase protein transcripts and basal neutrophil accumulation in the younger animals (Supplementary Fig. 5).

### 3.4 Activation of the hepatic acute phase response after intracerebral injection of 1 ng IL-1β reduces signs of injury and neutrophil recruitment to the brain

**Fig. 4:**
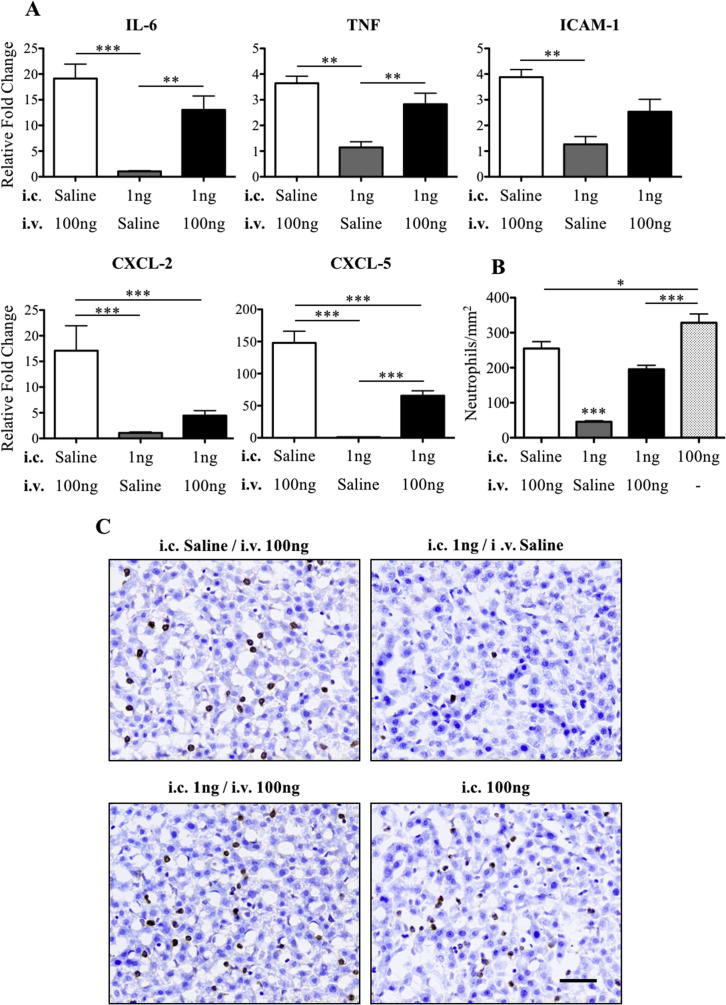
Activation of the acute phase response after intracerebral injection of 1 ng IL-1β at P14 by intravenous injection of 100 ng IL-1β. (A) Liver gene expression of inflammatory cytokines and chemokines, IL-6, TNF, ICAM-1, CXCL-2 and CXCL-5 at 4 h in three different inflammatory paradigms: saline i.c. + 100 ng i.v., 1 ng i.c. + saline i.v. and 1 ng i.c. + 100 ng i.v. Data are represented as relative fold change from expression levels in the 1 ng i.c. + saline i.v. group of animals. The intravenous administration of 100 ng IL-1β significantly increased expression of pro-inflammatory cytokines within the liver in comparison to the 1 ng IL-1β intracerebral injection alone. The combined central and systemic IL-1β administration significantly reduced expression of CXCL-2 and CXCL-5 compared to systemic IL-1β alone. Neutrophil numbers in the liver of P14 mice that received an i.v. injection of 100 ng IL-1β or the combined treatment of i.v. 100 ng IL-1β and i.c. 1 ng IL-1β were significantly higher than in animals that received an i.c. injection of 1 ng IL-1β alone (B, C). The number of neutrophils found in the liver following the i.c. injection of 100 ng IL-1β alone was significantly higher than all other groups (B, C). Scale bar = 50 µm. ^*^p < .05, ^**^p < .01 and ^***^p < .001. i.v.: intravenous, i.c.: intracerebral. saline i.c. + 100 ng i.v.: n = 3, 1 ng i.c. + saline i.v.: n = 8, 1 ng i.c./100 ng i.v.: n = 8 and 100 ng i.c.: n = 6.

The previous results showed that there is an inverse relationship between the severity of the cellular recruitment and blood–brain barrier permeability, and the magnitude of the hepatic APR at P14. This observation led us to hypothesise that activation of the peripheral APR may actually inhibit neutrophil recruitment to the brain and blood–brain barrier breakdown. In order to test this hypothesis, an APR was induced with an intravenous injection of 100 ng IL-1β immediately after intracerebral injection of 1 ng IL-1β. Data from these animals was compared to mice that received intracerebral injection of 1 ng IL-1β and intravenous injection of saline or intracerebral injection of saline and intravenous injections of 100 ng IL-1β. The APR was significantly induced in the livers of mice that received 100 ng of intravenous IL-1β (Fig. 4A). Interestingly, when this was coupled to an intracerebral injection of 1 ng IL-1β, the magnitude of the response was reduced for all transcripts (though only statistically significantly for CXCL-2/5, p < .001). Thus, the 1 ng intracerebral injection seems to at least partially inhibit the APR. Both 100 ng intravenous or intracerebral IL-1β injections resulted in a significant accumulation of neutrophils within the liver. By comparison, activation of the APR by intravenous IL-1β following the 1 ng intracerebral injection resulted in a relative reduction in neutrophils (statistically different from the response with high dose intracerebral IL-1β, p < .001) (Fig. 4B and C).

**Fig. 5:**
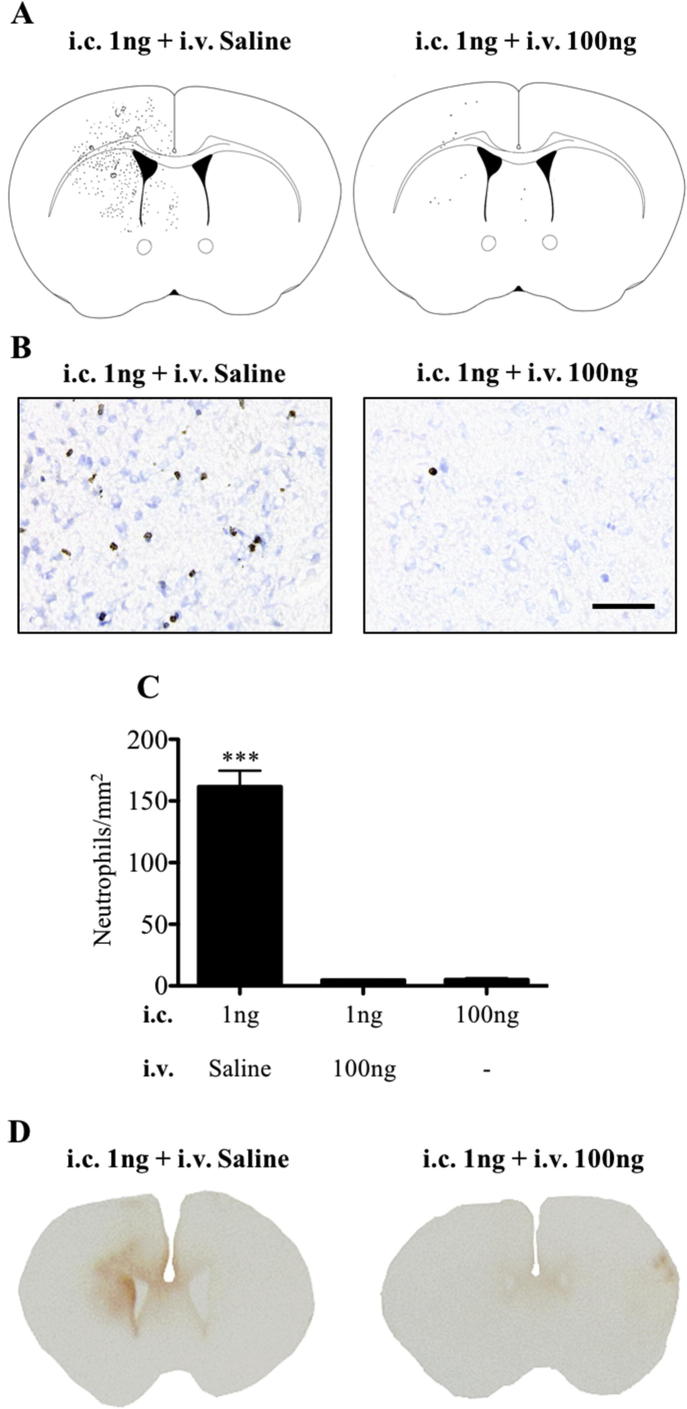
Modulation of neutrophil recruitment to the brain and blood–brain barrier permeability by activation of the systemic acute phase response at P14. Combined administration of i.c. 1 ng IL-1β and i.v. 100 ng IL-1β resulted in reduced neutrophil recruitment to the brain at 4 h as shown by representative spatial maps of neutrophil distribution in the brain (A) and photomicrographs of immunohistochemistry for neutrophils (brown, anti-neutrophil serum counterstained with cresyl violet) (B). The response was significantly less than when only 1 ng of IL-1β was injected i.c., and was the same as when 100 ng of IL-1β was injected i.c. (C). Representative pictures of blood–brain barrier permeability to endogenous IgG in the 1 ng i.c. + saline or 100 ng i.v. groups show that the combined treatment also reduces blood–brain barrier permeability (D). Scale bar = 50 µm. ^***^p < .001. i.v.: intravenous, i.c.: intracerebral. 1 ng i.c./saline i.v.: n = 3, 1 ng i.c./100 ng i.v.: n = 4 and 100 ng i.c.: n = 6. (For interpretation of the references to colour in this figure legend, the reader is referred to the web version of this article.)

The impact of the intravenous injection of 100 ng IL-1β on the inflammatory response to intracerebral injection of 1 ng IL-1β on the window of susceptibility at P14 was profound. The number of neutrophils present in the striatum was significantly decreased (4 ± 0.4 cells/mm^2^, p < .001) in the animals that received 100 ng IL-1β intravenously after intracerebral injection of 1 ng IL-1β compared to those that received an intracerebral injection of 1 ng IL-1β followed by an intravenous injection of saline (162 ± 13 cells/mm^2^). Indeed, the level of recruitment was reduced to the same number induced by an intracerebral injection of 100 ng IL-1β (5 ± 1 cells/mm^2^) (Fig. 5B and C). Blood-brain barrier permeability to endogenous IgG was decreased in mice that received intravenous injection of 100 ng IL-1β when compared to those that received intravenous injection of saline after 1 ng IL-1β injection in the brain (Fig. 5D). Surprisingly, brain mRNA expression for IL-1β and ICAM-1 in the striatum did not change after intravenous injection of 100 ng IL-1β compared to mice that received intravenous injection of saline after 1 ng IL-1β injection in the brain (Supplementary Fig. 6). Thus, the local production of inflammatory mediators in the brain was unaffected by the peripheral challenge.

## 4 Discussion

We have used a well-characterized model of brain inflammation (Anthony et al., 1997, Campbell et al., 2007a, Campbell et al., 2007b, Campbell et al., 2003, Campbell et al., 2002, Davis et al., 2005, Docagne et al., 2005, Sibson et al., 2004, Wilcockson et al., 2002) to study the cross-talk between brain and liver during postnatal period. Many studies have shown that a stereotaxic injection of IL-1β into the striatum of adult rodents induces the hepatic APR, which in turn, contributes to the increased recruitment of neutrophils/monocytes to the brain (Campbell et al., 2008, Campbell et al., 2005, Campbell et al., 2003, Wilcockson et al., 2002). It has been established that the juvenile rodent brain is more susceptible to the intracerebral injection of IL-1β when compared to adults and neonates, with substantial neutrophil recruitment observed from 4 h and extending for days following focal inflammation (Anthony et al., 1997, Blamire et al., 2000, Campbell et al., 2007a, Campbell et al., 2007b).

Our results show that the activation of an APR in the perinatal period inhibits, rather than augments, the response to acute brain inflammation, and is counterintuitive when considered in light of similar studies in adult animals. Overall, our findings suggest that the suppression of the peripheral inflammatory response after perinatal brain injury might increase rather than decrease the CNS inflammatory response and thus outcomes to injury.

### 4.1 Brain susceptibility to focal inflammation is age and dose-dependent

In our study, a window of susceptibility to a low dose of IL-1β (1ng) was found to peak at P14 and was not present at P7, P21 or P56. In rats, a similar window has been described at P21 and was considered to be associated with weaning or increased oligodendrocyte migration (Anthony et al., 1997). However, P14 in mice is in the pre-weaning period and myelination peaks at P20 in both rats and mice (Semple et al., 2013). Thus, the biology underpinning this susceptible period must lie elsewhere. Lawson and Perry (1995) also showed that LPS injected in the striatum of BALB/c mice promoted an increased neutrophil recruitment to the brain at P7 and not at P0. However, they did not study any further time points and appear to have missed the P14 window. An LPS injection into Sprague-Dawley rats transiently increases blood–brain barrier permeability in the first postnatal week (Stolp et al., 2005). Increased neutrophil recruitment to the brain and altered blood–brain barrier permeability has also been reported following the combination of systemic LPS administration and central hypoxic-ischemic injury at P12 but not at P1 in Lewis rats (Brochu et al., 2011). While there are differences in the specific timing of injury in these studies, likely due to the variation in immunogen, species and study design, they all show the same phenomena of a specific period of postnatal rodent development which is acutely susceptible to altered blood–brain barrier permeability as a result of inflammation.

It is our hypothesis that the increase in blood–brain barrier permeability to IgG at P14 is likely to be driven by the extensive neutrophil recruitment at this age and neutrophil depletion prevents cytokine-induced blood–brain barrier breakdown. Anthony et al., 1997, Anthony et al., 1998 showed that the acute increase in blood–brain barrier permeability produced at P21 in their rat model (either with IL-1β or CXCL-1) was neutrophil-dependent, and also associated with disruption of tight junction proteins (Bolton et al., 1998). Neutrophils produce high levels of reactive oxygen species that have been hypothesized to affect blood–brain barrier integrity by activation of matrix metalloproteinases, oxidative damage, modulation of tight junction proteins, cytoskeletal reorganization, and induction of inflammatory mediators (Pun et al., 2009). Matrix metalloproteinase 9 released by neutrophils may also digest vessel basement membrane and/or extracellular matrix and, consequently, contribute to the increased permeability of the blood–brain barrier (Justicia et al., 2003, Moxon-Emre and Schlichter, 2011, Rosell et al., 2008, Turner and Sharp, 2016, Justicia et al., 2003, Moxon-Emre and Schlichter, 2011, Rosell et al., 2008, Turner and Sharp, 2016), providing a potential mechanism for the neutrophil dependence of this phenomenon.

Here, uniquely, we observed that the window of susceptibility was dose dependent at P14, with significantly fewer neutrophils being recruited in response to a rising dose of IL-1β and a reduction in blood–brain barrier permeability at the 100 ng dose. However, at P56 this dose resulted in a small, but significant increase in neutrophil recruitment, which did not coincide with a disruption of the blood–brain barrier to endogenous IgG. The atypical nature of the CNS response to pro-inflammatory challenges is widely reported though the mechanism is still unknown (Campbell et al., 2007a, Campbell et al., 2007b).

### 4.2 The hepatic acute phase response to cerebral focal inflammation

Inflammation in the CNS is known to be followed by a peripheral APR that is coordinated mainly by the liver in adult rodents. Stereotaxic injection of TNF (1000 ng) or IL-1β (1–1000 ng) in the striatum of adult rats induced a hepatic APR, observed as an upregulation of the expression of chemokines and increased recruitment of leukocytes, including neutrophils, 4 h after brain injection (Campbell et al., 2005, Campbell et al., 2003). Stereotaxic injection of IL-1β (1ng) in the striatum has been shown to induce the expression of acute phase proteins, including serum amyloid A and P, in the liver 6 h after the injection in adult C57Bl/6 mice (Wilcockson et al., 2002). The presence of inflammation in the CNS has recently been shown to be transmitted to the periphery via astrocyte-shed extracellular vesicles that activate the production of acute phase proteins (Dickens et al., 2017).

In this study, the liver only produced a consistent and significant APR following the injection of a high dose of IL-1β into the brain. The response was seen at all ages examined, but there was an age-specific difference in the magnitude of this response. Our own experiments have shown that IL-1β injected into the brain parenchyma does not leave the brain (Couch et al., 2017) and the effect of the combination of the 1 ng intracerebral dose with a 100 ng intravenous dose compared to the 100 ng intravenous alone suggests that simple leakage from the brain is not a factor here. Indeed, the presence of 1 ng IL-1β in the brain seems to inhibit the APR in these young animals and the local production of IL-1β and ICAM-1 was unaffected by high circulating concentrations of IL-1β.

The hepatic immune response in young animals is understudied, adding to the novelty of the current work. Bonestroo et al. (2013) have shown that cerebral hypoxia–ischemia caused a hepatic APR after 3 h in P7 rats characterized by upregulation of CXCL-1 and downregulation of TNF, IL-1β and CCL-2. However, this study did not explore the hepatic response in other postnatal ages or in adults for a developmental comparison. The higher magnitude of the response in the younger animals observed in the present study may be due to the innately elevated immune status of the liver at these ages. In rodents, the liver undergoes an extensive structural remodelling during the first four weeks after birth (Alexander et al., 1997, Walthall et al., 2005), and the liver weight increases as a result of the hepatic cell proliferation (Apte et al., 2007, Behrens et al., 2002, Septer et al., 2012). TNF and IL-1 have been shown to have an important role in hepatocytes growth during this postnatal period by activating the NF-kB pathway (Kinoshita and Miyajima, 2002, May and Ghosh, 1999). Thus, the high expression of cytokines observed in our study may be explained by their important role in liver development. Consequently, the expression of cytokines in the liver decreases as this organ maturates.

Increased expression of inflammatory mediators in the liver early in postnatal period may also be directly linked to the increased number of neutrophils. Neutrophils start to appear in the liver at E15 and their number increases until P0, where they remain proliferative in small distributed foci (Ayres-Silva et al., 2011), which was also observed at P7 in our study. As a consequence of liver maturation, mature or immature neutrophils leave the liver as the environment ceases to be favourable (Ayres-Silva et al., 2011, Kinoshita et al., 1999, Si-Tayeb et al., 2010), and neutrophils are largely absent from the adult healthy liver (Campbell et al., 2003, Gregory et al., 1996). This may follow from a reduction in hepatic chemokine production in the postnatal period, but as neutrophils also express a number of CXC and CC chemokines it is hard to determine whether the decrease is simply the result of a reduction in cell number. It is unclear whether there is a direct role of adhesion molecules, chemokines and neutrophils in liver development, however, ICAM-1 and neutrophils were shown to have a crucial role in liver regeneration after partial hepatectomy; this may indicate its importance in liver growth during development. Selzner et al. (2003) have suggested that the expression of ICAM-1 in Kupffer cells and endothelial cells after partial hepatectomy promotes neutrophils recruitment, which activates the release of IL-6 and TNF by the Kupffer cells to promote regeneration. As a result of these developmental phenomena, the postnatal rodent liver may be primed to respond to inflammatory challenge.

### 4.3 Hepatic acute phase response can protect the brain from excessive neutrophil recruitment and altered blood–brain barrier permeability

The local inflammatory response to intracerebral injection of IL-1β was related to the dose administered, with the expression of IL-1β and ICAM-1 in the brain positively correlated with the concentration of IL-1β injected. However, the neutrophil recruitment and increased blood–brain barrier permeability to IgG was inversely affected and we wondered how this phenomenon, which could not have been predicted from previous work in adult animals, might be related to the APR. The APR in the liver has been correlated with the most severe cases of ischemic stroke and with traumatic brain injury (Di Napoli et al., 2005, Smith et al., 2006, Villapol, 2016). Previous studies have also shown that stereotaxic injection of TNF or IL-1β in adult rats not only induced the APR in the liver, but augmentation of the APR increased the recruitment of leukocytes to the brain (Campbell et al., 2005, Campbell et al., 2003). Consequently, Campbell et al., 2007a, Campbell et al., 2007b showed that the peripheral administration of etanercept, a competitive TNF inhibitor, reduced the recruitment of neutrophils to an IL-1β-induced focal inflammatory lesion in the brain via inhibition of the hepatic APR in adult rats. Cerebral hypoxia–ischemia in P7 rats also increased CXCL-1 expression in the liver and recruitment of neutrophils to the affected brain hemisphere (Bonestroo et al., 2013). The timing and type of injury, as well as the animal species, may underlie differences between the study of Bonestroo et al. (2013) and our results. We have shown that while the number of neutrophils in the brain parenchyma decreased at P7, P14 and P21 with higher doses of IL-1β, at P56 there was an increase in neutrophils in the brain under the same circumstances. This suggests that the observed inverse relationship between the severity of brain cellular injury and the magnitude of the APR in the liver is a specific developmental process. It is important to note that the APR to an intracerebral injection of 100 ng IL-1β was much higher at P14 than in the adult. This may be, in part, due to the increased basal levels of inflammatory mediators at the early time points and the increased number of basal neutrophils within the P14 naïve liver. Thus, it seems that the liver may be primed at the time of inflammation in the brain, and this 'head start' in the activation process may result in the increased magnitude of the APR at P14. As a consequence, it is possible that the peripheral response then overwhelms the inflammatory signals generated locally in the brain and, thus, neutrophils are redirected to the liver and possibly to other peripheral organs. In the adult, the APR is smaller and, thus, may not out-compete the local injury signals in the brain that recruit neutrophils (albeit a small number at acute time points).

The inverse relationship between magnitude of cellular inflammatory responses in the brain and liver observed at P14 also suggests that activation of the APR may protect the brain from excessive neutrophil recruitment and blood–brain barrier permeability. Indeed, by injecting 100 ng IL-1β intravenous immediately after stereotaxic injection of 1 ng IL-1β in the striatum of P14 mice, the hepatic response was activated (increased neutrophils recruitment and expression of inflammatory cytokines and chemokines) and, consequently, eliminated almost all neutrophils from the brain without inducing the expression of IL-1β and ICAM-1 in the brain. These results support our hypothesis and showed, for the first time, a protective role of the APR in the periphery to focal inflammation during a specific window of development. Moreover, they suggest that experimental activation of the peripheral immune response may be used to reduce neonatal brain injury. It is important to note that while these findings illuminate a novel developmental response to inflammatory modulation, the outcomes assessed are acute and long-term effects on both the inflammatory response and neuropathology need to be determined before this work can be translated to a novel therapeutic approach.

Many anti-inflammatory therapies/drugs have been shown to be beneficial for disorders in the adulthood, such as acute stroke and traumatic brain injury, with an inflammatory component (Bergold, 2016, Smith et al., 2015) while use of the steroid-based anti-inflammatory agent dexamethasone in prenatal infants during postnatal period has been associated with growth deficits at 2 years (Rijken et al., 2007) and decreases in cerebral cortical grey matter volume at term time (Murphy et al., 2001) and later in life (Cheong et al., 2014). Our data supports the observation that pro-inflammatory agents may not be universally protective, and that a different, age-specific, therapeutic strategy needs to be explored for these children. While this study does not directly model a specific neuropathology, nor stage of human development, these findings highlight the importance of matching therapeutic paradigms to age and type of injury, and that treatment decisions should not be applied on the basis of normal adult biology, a lesson also important for the ageing brain (Campbell et al., 2007a, Campbell et al., 2007b).

In the future, it will be important to further study the relationship between the brain and the hepatic inflammatory responses in different inflammatory contexts. In particular, the effects of experimental activation of the APR after inflammatory injuries in the brain during development and in adults in order to confirm the age-dependent effects of the hepatic immune response to brain damage.

## 5 Conclusion

This study has demonstrated the importance of the interplay between the brain and peripheral immune responses in defining the magnitude of the acute inflammatory response in the brain to focal inflammation during postnatal development in mice. The data support the current view that susceptibility of the brain to inflammation varies with age and that the peripheral immune environment may define the overall response to injury. Activation of the APR in the liver was shown to invert the window of susceptibility in a mouse model of cerebral focal inflammation and, therefore, selective activation of the peripheral inflammatory response may be a therapeutic option at specific developmental stages. This work highlights the importance of selecting age-dependent therapy for acute brain injury.

## Figures and Tables

**Supplementary Fig. A1:**
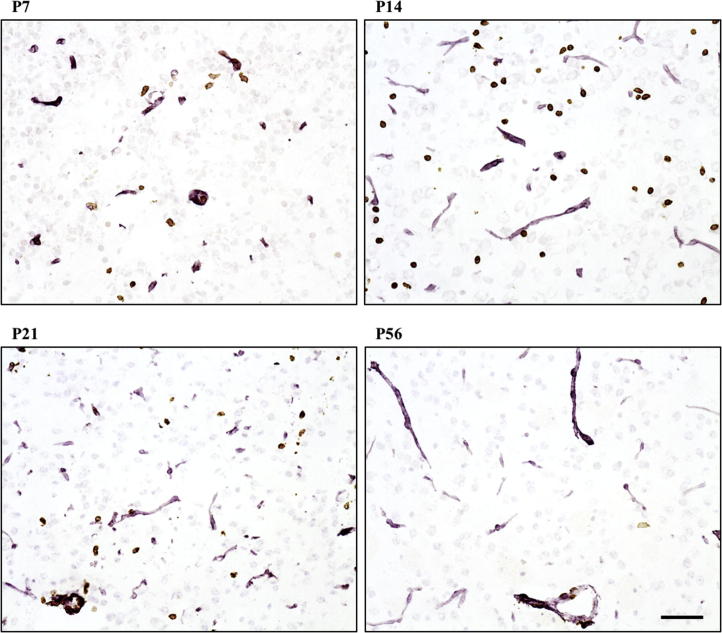
Recruitment of neutrophils to the brain 4 h after intracerebral injection of 1ng IL-1β during postnatal period. Photomicrographs show neutrophils (brown; anti-neutrophil serum) and vessels (pink; laminin) in the striatal injection site 4 h after the intracerebral injection of 1ng IL-1β at P7, P14, P21 and P56. These not only show that more neutrophils were present in the brain, but also that more cells were able to migrate into the parenchyma at P14. Scale bar = 50 µm.

**Supplementary Fig. A2:**
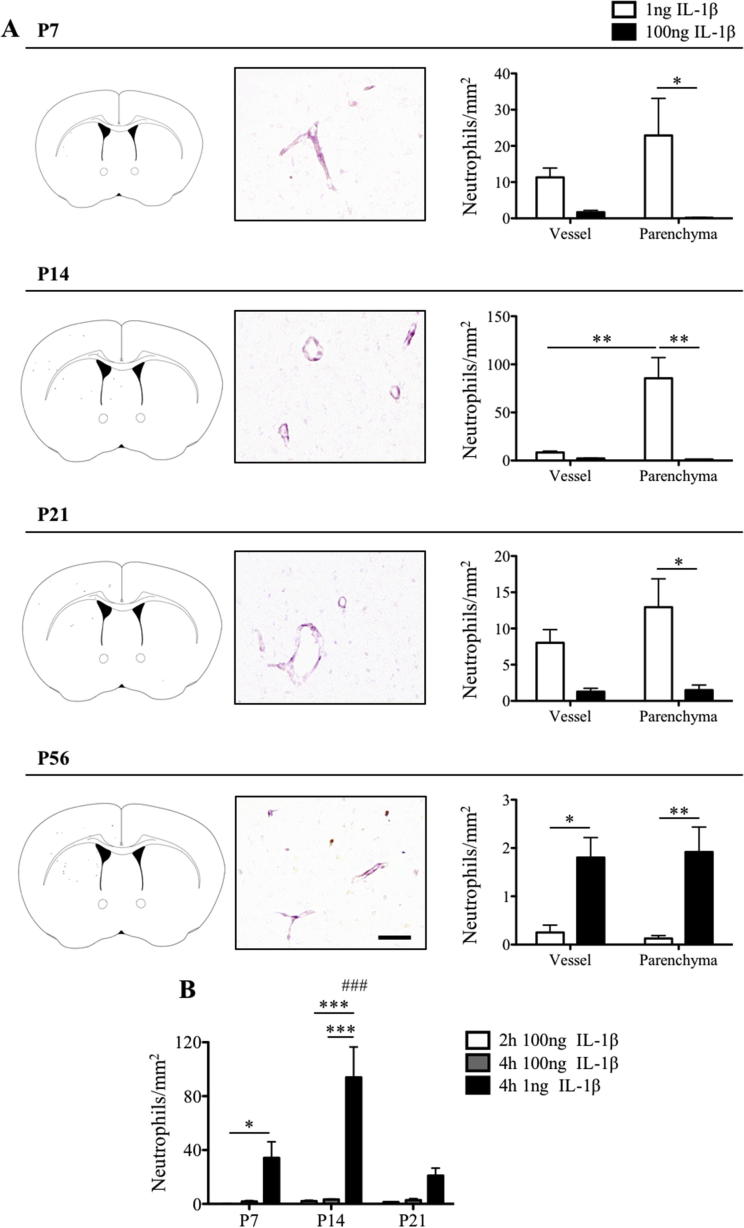
Recruitment of neutrophils to brain parenchyma after intracerebral injection of 100 ng IL-1β during postnatal period. Neutrophil distribution in the brain, shown by representative spatial maps and immunohistochemistry of neutrophils (brown; anti-neutrophil serum) and vessels (pink; laminin), was limited at 4 h following intracerebral injection of 100 ng IL-1β into the striatum at P7, P14, P21 and P56. The number of neutrophils per mm^2^ of striatum either in the parenchyma or in vessels was significantly lower after the 100 ng IL-1β intracerebral injection compared to the 1ng IL-1β injection at P7, P14, P21. In contrast, at P56, the number of neutrophils was significantly higher following the 100 ng compared to the 1ng dose of IL-1β (A). The absence of the window of susceptibility, observed at 4 h, was also absent at 2 h after intracerebral injection of 100 ng IL-1β at P7, P14 and P21 when compared to the 1ng dose at 4 h (neutrophils per mm^2^ of striatum after immunohistochemistry using anti-neutrophil serum counterstained with cresyl violet) (B). Scale bar = 50 µm. ^∗^p < .05, ^∗∗^p < .01, ^∗∗∗^p < .001 and ^###^p < 0.001 vs. all 4 h (h) 1ng values. 4 h — P7: n = 4 each group; P14: n = 5 for 1ng IL-1β, n = 3 for 100 ng IL-1β; P21: n = 4 for 1ng IL-1β, n = 3 for 100 ng IL-1β; P56: n = 3 each group; 2 h - P7: n = 6, P14: n = 6 and P21: n = 5.

**Supplementary Fig. A3:**
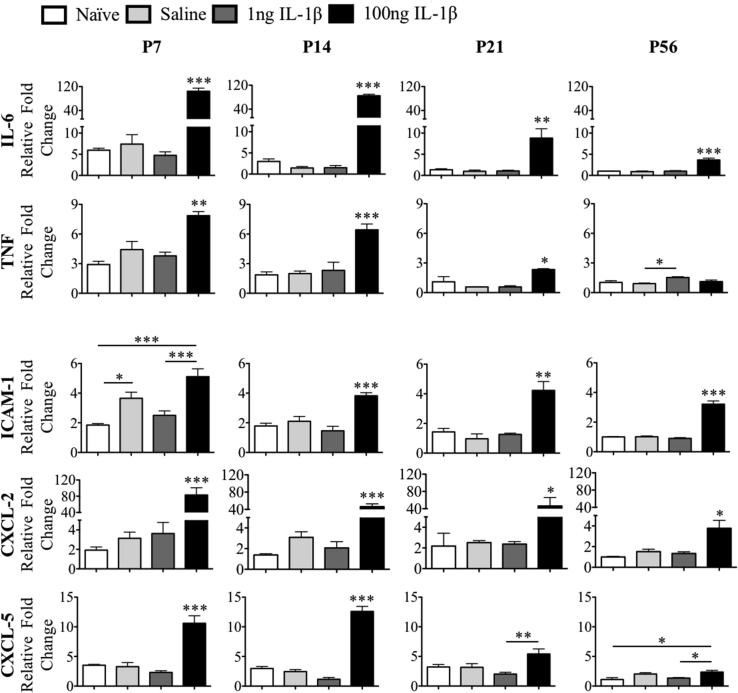
The expression of inflammatory mediators in the liver in response to a low or high dose of IL-1β in the brain during postnatal period. IL-6, TNF, ICAM-1, CXCL-2 and CXCL-5 mRNA expression in the liver of naïve mice and mice that received intracerebral injection of saline or 1ng IL-1β or 100 ng IL-1β at different postnatal ages (P7, P14, P21 and P56). Data are represented as relative fold change from expression levels of P56 mice. At all ages, there was a significant increase in liver gene expression of inflammatory molecules 4 h after the 100 ng intracerebral injection. The 1ng dose only caused an increase in gene expression at P56 for TNF. ^∗^p < 0.05, ^∗∗^p < 0.01 and ^∗∗∗^p < 0.001. P7: n = 4 for saline, n = 5 for 1ng and 100 ng IL-1β; P14: n = 4 for saline, n = 3 for 1ng IL-1β, n = 5 for 100 ng IL-1β, P21: n = 4 for saline, 1ng IL-1β and 100 ng IL-1β; P56: n = 4 for saline and 1ng IL-1β, n = 5 for 100 ng IL-1β.

**Supplementary Fig. A4:**
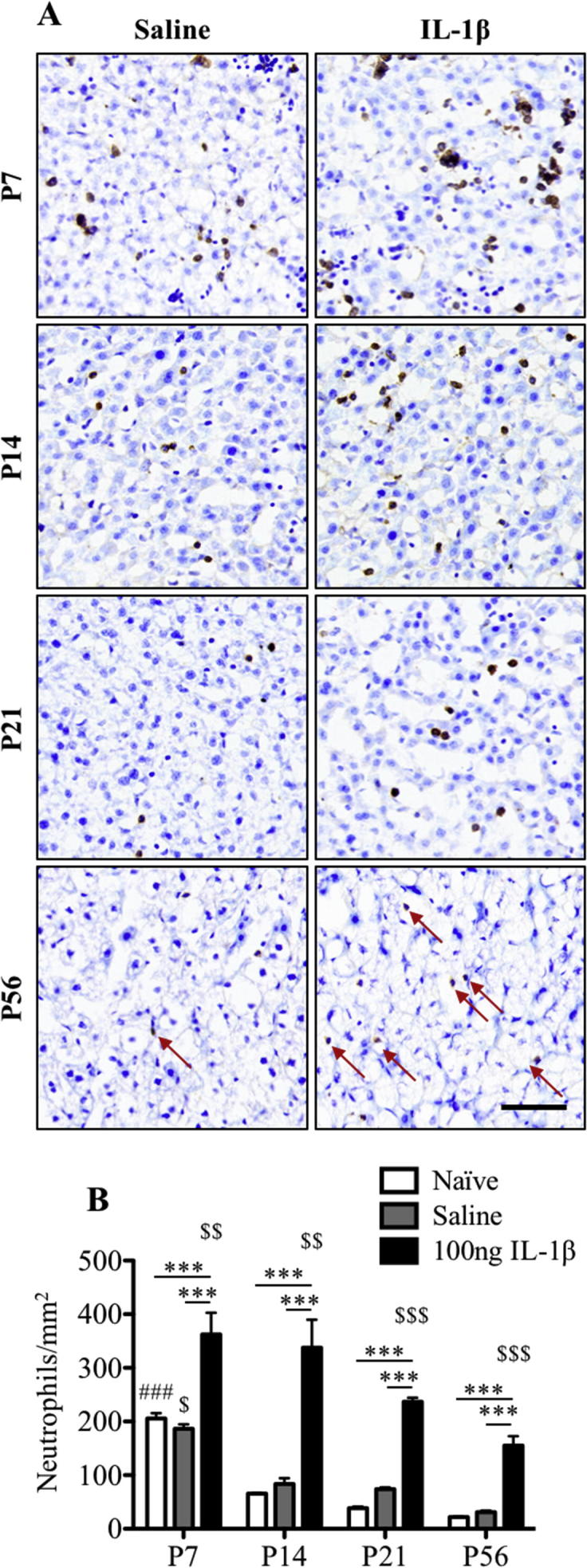
Neutrophil recruitment to the liver 4 h after intracerebral injection of 100 ng IL-1β. Immunohistochemistry for neutrophils revealed higher numbers of cells in the liver following intracerebral injection of 100 ng IL-1β at all ages compared to naïve or saline injected animals (A). The greatest response was observed in the youngest animals (B). Scale bar = 50 µm. ^∗∗^p < 0.01 and ^∗∗∗^p < 0.001. ^###^p < 0.001 vs. all naïve values, $ significantly different from all saline values, $$ significantly different from 100 ng P21 and P56 and $$$ significantly different from all 100 ng values. P7: n = 3 each group, P14: n = 3 each group, P21: n = 3 each group and P56: n = 4 for naïve and saline, n = 7 for 100 ng IL-1β.

**Supplementary Fig. A5:**
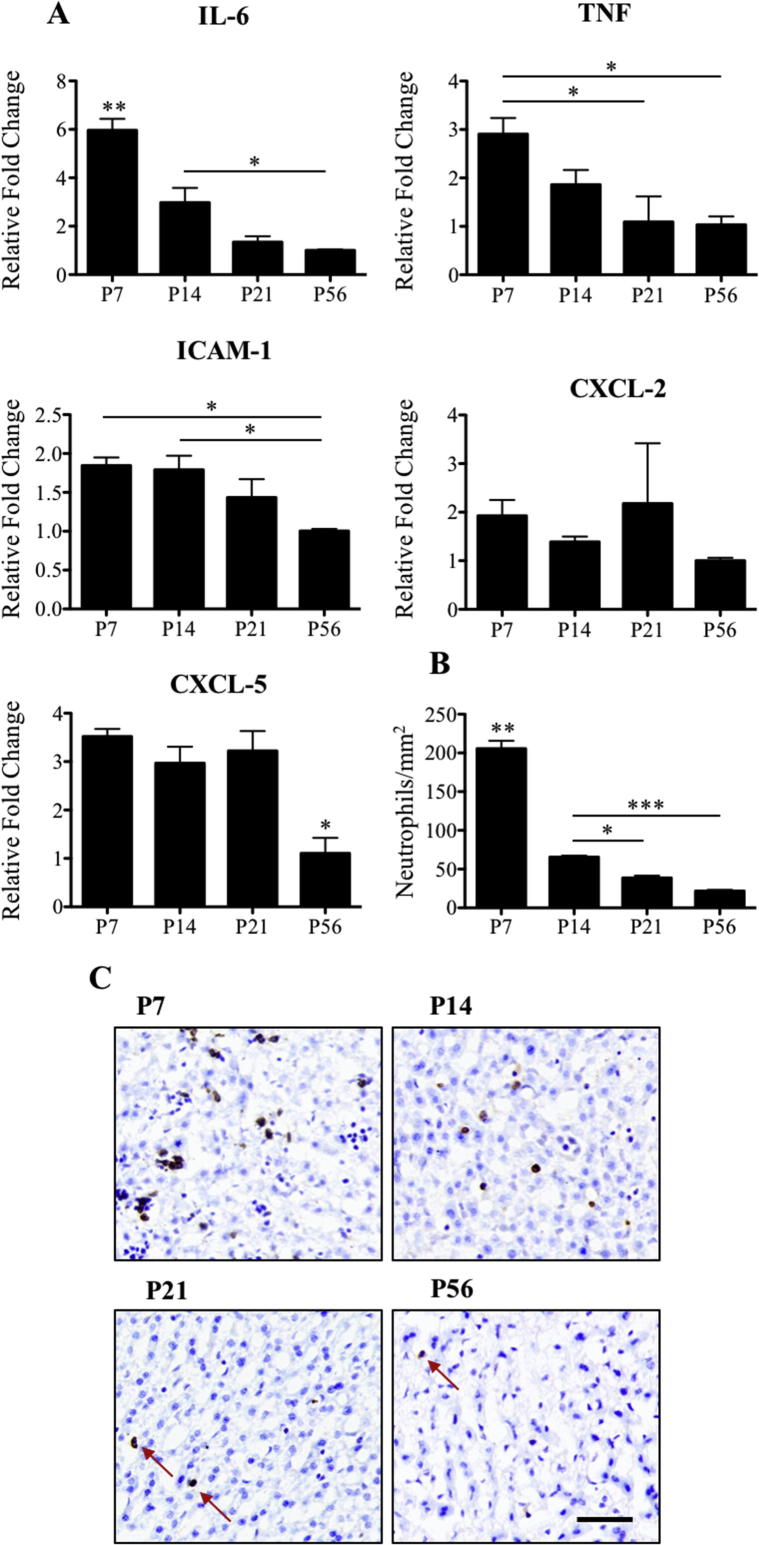
mRNA expression of inflammatory mediators and neutrophil population in the liver during postnatal development. (A) IL-6, TNF, ICAM-1, CXCL-2 and CXCL-5 mRNA expression in the liver of naïve mice at different postnatal ages (P7, P14, P21 and P56). Data are represented as relative fold change from expression levels of P56 mice. For most genes, the highest expression was measured at P7 and was reduced during further postnatal development. No change in CXCL-2 was found in the liver over the developmental period studied, while the expression of CXCL-5 remained high until P21 and was significantly reduced at P56. The number of neutrophils per mm^2^ of liver also decreased during postnatal development (B, C). Scale bar = 50 µm. ^∗^p < 0.05, ^∗∗^p < 0.01 and ^∗∗∗^p < 0.001. P7: n = 4, P14: n = 4, P21 n = 4 and P56: n = 3.

**Supplementary Fig. A6:**
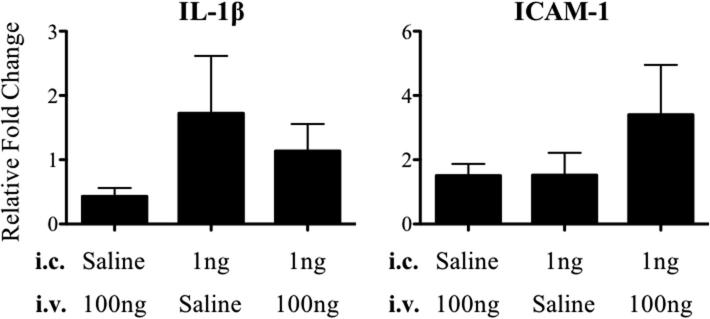
The effect of experimental activation of the acute phase response on the expression of inflammatory mediators in the striatum 4 h after injection of 1ng IL-1β in the brain. IL-1β and ICAM-1 mRNA expression in the ipsilateral striatum of three different groups of mice: saline i.c + 100 ng i.v., 1ng i.c + saline i.v. and 1ng i.c. + 100 ng i.v. Data are represented as relative fold change from expression levels of 1ng i.c + saline i.v. group of mice. Note: no significant difference in inflammatory gene expression was observed in the brain in these treatment groups, in contrast to the large difference in gene expression observed in the liver. i.v.: intravenous, i.c.: intracerebral. Saline i.c./100 ng i.v.: n = 3, 1ng i.c./saline i.v.: n = 4 and 1ng i.c./100 ng i.v.: n = 4.

## References

[b0005] Acalovschi D., Wiest T., Hartmann M., Farahmi M., Mansmann U., Auffarth G.U., Grau A.J., Green F.R., Grond-Ginsbach C., Schwaninger M. (2003). Multiple levels of regulation of the interleukin-6 system in stroke. Stroke.

[b0010] Alexander B., Guzail M.A., Foster C.S. (1997). Morphological changes during hepatocellular maturity in neonatal rats. Anat. Rec..

[b0015] Anthony D., Dempster R., Fearn S., Clements J., Wells G., Perry V.H., Walker K. (1998). CXC chemokines generate age-related increases in neutrophil-mediated brain inflammation and blood–brain barrier breakdown. Curr. Biol..

[b0020] Anthony D.C., Bolton S.J., Fearn S., Perry V.H. (1997). Age-related effects of interleukin-1 beta on polymorphonuclear neutrophil-dependent increases in blood-brain barrier permeability in rats. Brain.

[b0025] Anthony D.C., Couch Y. (2014). The systemic response to CNS injury. Exp. Neurol..

[b0030] Anthony D.C., Couch Y., Losey P., Evans M.C. (2012). The systemic response to brain injury and disease. Brain Behav. Immun..

[b0035] Apte U., Zeng G., Thompson M.D., Muller P., Micsenyi A., Cieply B., Kaestner K.H., Monga S.P.S. (2007). beta-Catenin is critical for early postnatal liver growth. AJP Gastrointest. Liver Physiol..

[b0040] Atladóttir H.Ó., Thorsen P., Østergaard L., Schendel D.E., Lemcke S., Abdallah M., Parner E.T. (2010). Maternal infection requiring hospitalization during pregnancy and autism spectrum disorders. J. Autism Dev. Disord..

[b0045] Ayres-Silva Jde P., Manso P.P., Madeira M.R., Pelajo-Machado M., Lenzi H.L. (2011). Sequential morphological characteristics of murine fetal liver hematopoietic microenvironment in Swiss Webster mice. Cell Tissue Res..

[b0050] Bayer T.A., Buslei R., Havas L., Falkai P. (1999). Evidence for activation of microglia in patients with psychiatric illnesses. Neurosci. Lett..

[b0055] Behrens A., Sibilia M., David J.-P., Möhle-Steinlein U., Tronche F., Schütz G., Wagner E.F. (2002). Impaired postnatal hepatocyte proliferation and liver regeneration in mice lacking c-jun in the liver. EMBO J..

[b0060] Bergold P.J. (2016). Treatment of traumatic brain injury with anti-inflammatory drugs. Exp. Neurol..

[b0065] Blamire A.M., Anthony D.C., Rajagopalan B., Sibson N.R., Perry V.H., Styles P. (2000). Interleukin-1beta-induced changes in blood-brain barrier permeability, apparent diffusion coefficient, and cerebral blood volume in the rat brain: a magnetic resonance study. J. Neurosci..

[b0070] Blond D., Campbell S.J., Butchart A.G., Perry V.H., Anthony D.C. (2002). Differential induction of interleukin-1beta and tumour necrosis factor-alpha may account for specific patterns of leukocyte recruitment in the brain. Brain Res..

[b0075] Bloomfield P.S., Selvaraj S., Veronese M., Rizzo G., Bertoldo A., Owen D.R., Bloomfield M.A.P., Bonoldi I., Kalk N., Turkheimer F., McGuire P., de Paola V., Howes O.D. (2016). Microglial activity in people at ultra high risk of psychosis and in schizophrenia: an [^11^C]PBR28 PET Brain Imaging Study. Am. J. Psychiatry.

[b0080] Bolton S.J., Anthony D.C., Perry V.H. (1998). Loss of the tight junction proteins occludin and zonula occludens-1 from cerebral vascular endothelium during neutrophil-induced blood-brain barrier breakdown in vivo. Neuroscience.

[b0085] Bonestroo H.J.C., Nijboer C.H.A., van Velthoven C.T.J., Kavelaars A., Hack C.E., van Bel F., Heijnen C.J. (2013). Cerebral and hepatic inflammatory response after neonatal hypoxia-ischemia in newborn rats. Dev. Neurosci..

[b0090] Brochu M.-E., Girard S., Lavoie K., Sébire G. (2011). Developmental regulation of the neuroinflammatory responses to LPS and/or hypoxia-ischemia between preterm and term neonates: an experimental study. J. Neuroinflamm..

[b0095] Brown A.S., Begg M.D., Gravenstein S., Schaefer C.A., Wyatt R.J., Bresnahan M., Babulas V.P., Susser E.S. (2004). Serologic Evidence of prenatal influenza in the etiology of schizophrenia. Arch. Gen. Psychiatry.

[b0100] Campbell S.J., Anthony D.C., Oakley F., Carlsen H., Elsharkawy A.M., Blomhoff R., Mann D.A. (2008). Hepatic nuclear factor kappa B regulates neutrophil recruitment to the injured brain. J. Neuropathol. Exp. Neurol..

[b0105] Campbell S.J., Carare-Nnadi R.O., Losey P.H., Anthony D.C. (2007). Loss of the atypical inflammatory response in juvenile and aged rats. Neuropathol. Appl. Neurobiol..

[b0110] Campbell S.J., Hughes P.M., Iredale J.P., Wilcockson D.C., Waters S., Docagne F., Perry V.H., Anthony D.C. (2003). CINC-1 is an acute-phase protein induced by focal brain injury causing leukocyte mobilization and liver injury. FASEB J..

[b0115] Campbell S.J., Jiang Y., Davis A.E.M., Farrands R., Holbrook J., Leppert D., Anthony D.C. (2007). Immunomodulatory effects of etanercept in a model of brain injury act through attenuation of the acute-phase response. J. Neurochem..

[b0120] Campbell S.J., Perry V.H., Pitossi F.J., Butchart A.G., Chertoff M., Waters S., Dempster R., Anthony D.C. (2005). Central nervous system injury triggers hepatic CC and CXC chemokine expression that is associated with leukocyte mobilization and recruitment to both the central nervous system and the liver. Am. J. Pathol..

[b0125] Campbell S.J., Wilcockson D.C., Butchart A.G., Perry V.H., Anthony D.C. (2002). Altered chemokine expression in the spinal cord and brain contributes to differential interleukin-1beta-induced neutrophil recruitment. J. Neurochem..

[b0130] Catroppa C., Anderson V.A., Morse S.A., Haritou F., Rosenfeld J.V. (2008). Outcome and predictors of functional recovery 5 years following pediatric traumatic brain injury (TBI). J. Pediatr. Psychol..

[b0135] Chapman K.Z., Dale V.Q., Dénes A., Bennett G., Rothwell N.J., Allan S.M., McColl B.W. (2009). A rapid and transient peripheral inflammatory response precedes brain inflammation after experimental stroke. J. Cereb. Blood Flow Metab..

[b0140] Cheong J.L.Y., Burnett A.C., Lee K.J., Roberts G., Thompson D.K., Wood S.J., Connelly A., Anderson P.J., Doyle L.W., Victorian Infant Collaborative Study Group (2014). Association between postnatal dexamethasone for treatment of bronchopulmonary dysplasia and brain volumes at adolescence in infants born very preterm. J. Pediatr..

[b0145] Couch Y., Akbar N., Roodselaar J., Evans M.C., Gardiner C., Sargent I., Romero I.A., Bristow A., Buchan A.M., Haughey N., Anthony D.C. (2017). Circulating endothelial cell-derived extracellular vesicles mediate the acute phase response and sickness behaviour associated with CNS inflammation. Sci. Rep..

[b0150] Couch Y., Davis A.E., Sá-Pereira I., Campbell S.J., Anthony D.C. (2014). Viral pre-challenge increases central nervous system inflammation after intracranial interleukin-1β injection. J. Neuroinflamm..

[b0155] Davis A.E.M., Campbell S.J., Wilainam P., Anthony D.C. (2005). Post-conditioning with lipopolysaccharide reduces the inflammatory infiltrate to the injured brain and spinal cord: a potential neuroprotective treatment. Eur. J. Neurosci..

[b0160] Di Napoli M., Schwaninger M., Cappelli R., Ceccarelli E., Di Gianfilippo G., Donati C., Emsley H.C.A., Forconi S., Hopkins S.J., Masotti L., Muir K.W., Paciucci A., Papa F., Roncacci S., Sander D., Sander K., Smith C.J., Stefanini A., Weber D. (2005). Evaluation of C-reactive protein measurement for assessing the risk and prognosis in ischemic stroke: a statement for health care professionals from the CRP Pooling Project members. Stroke.

[b9005] Dickens A.M., Tovar-Y-Romo L.B., Yoo S.W., Trout A.L., Bae M., Kanmogne M., Megra B., Williams D.W., Witwer K.W., Gacias M., Tabatadze N., Cole R.N., Casaccia P., Berman J.W., Anthony D.C., Haughey N.J. (2017). Astrocyte-shedextracellular vesicles regulate the peripheral leukocyte response toinflammatory brain lesions. Sci Signal.

[b0165] Docagne F., Campbell S.J., Bristow A.F., Poole S., Vigues S., Guaza C., Perry V.H., Anthony D.C. (2005). Differential regulation of type I and type II interleukin-1 receptors in focal brain inflammation. Eur. J. Neurosci..

[b0170] Dubowitz L.M., Bydder G.M., Mushin J. (1985). Developmental sequence of periventricular leukomalacia. Correlation of ultrasound, clinical, and nuclear magnetic resonance functions. Arch. Dis. Child..

[b0175] Duval J., Braun C.M.J., Montour-Proulx I., Daigneault S., Rouleau I., Bégin J. (2008). Brain lesions and IQ: recovery versus decline depends on age of onset. J. Child Neurol..

[b0180] Estes M.L., McAllister A.K. (2015). Immune mediators in the brain and peripheral tissues in autism spectrum disorder. Nat. Rev. Neurosci..

[b0185] Fillman S.G., Cloonan N., Catts V.S., Miller L.C., Wong J., McCrossin T., Cairns M., Weickert C.S. (2013). Increased inflammatory markers identified in the dorsolateral prefrontal cortex of individuals with schizophrenia. Mol. Psychiatry.

[b0190] Gregory S.H., Sagnimeni A.J., Wing E.J. (1996). Bacteria in the bloodstream are trapped in the liver and killed by immigrating neutrophils. J. Immunol..

[b0195] Justicia C., Panés J., Solé S., Cervera A., Deulofeu R., Chamorro A., Planas A.M. (2003). Neutrophil infiltration increases matrix metalloproteinase-9 in the ischemic brain after occlusion/reperfusion of the middle cerebral artery in rats. J. Cereb. Blood Flow Metab..

[b0200] Kadhim H., Tabarki B., De Prez C., Sébire G. (2003). Cytokine immunoreactivity in cortical and subcortical neurons in periventricular leukomalacia: are cytokines implicated in neuronal dysfunction in cerebral palsy?. Acta Neuropathol..

[b0205] Kadhim H., Tabarki B., Verellen G., De Prez C., Rona A.M., Sébire G. (2001). Inflammatory cytokines in the pathogenesis of periventricular leukomalacia. Neurology.

[b0210] Kinoshita T., Miyajima A. (2002). Cytokine regulation of liver development. Biochim. Biophys. Acta.

[b0215] Kinoshita T., Sekiguchi T., Xu M.J., Ito Y., Kamiya A., Tsuji K., Nakahata T., Miyajima A. (1999). Hepatic differentiation induced by oncostatin M attenuates fetal liver hematopoiesis. Proc. Natl. Acad. Sci. U.S.A..

[b0220] Lawson L.J., Perry V.H. (1995). The unique characteristics of inflammatory responses in mouse brain are acquired during postnatal development. Eur. J. Neurosci..

[b0225] Mallard C., Davidson J.O., Tan S., Green C.R., Bennet L., Robertson N.J., Gunn A.J. (2014). Astrocytes and microglia in acute cerebral injury underlying cerebral palsy associated with preterm birth. Pediatr. Res..

[b0230] May M.J., Ghosh S. (1999). IkappaB kinases: kinsmen with different crafts. Science.

[b0235] Mednick S.A., Machon R.A., Huttunen M.O., Bonett D. (1988). Adult schizophrenia following prenatal exposure to an influenza epidemic. Arch. Gen. Psychiatry.

[b0240] Meyer U., Nyffeler M., Engler A., Urwyler A., Schedlowski M., Knuesel I., Yee B.K., Feldon J. (2006). The time of prenatal immune challenge determines the specificity of inflammation-mediated brain and behavioral pathology. J. Neurosci..

[b0245] Moxon-Emre I., Schlichter L.C. (2011). Neutrophil depletion reduces blood-brain barrier breakdown, axon injury, and inflammation after intracerebral hemorrhage. J. Neuropathol. Exp. Neurol..

[b0250] Murphy B.P., Inder T.E., Huppi P.S., Warfield S., Zientara G.P., Kikinis R., Jolesz F.A., Volpe J.J. (2001). Impaired cerebral cortical gray matter growth after treatment with dexamethasone for neonatal chronic lung disease. Pediatrics.

[b0255] Pun P.B.L., Lu J., Moochhala S. (2009). Involvement of ROS in BBB dysfunction. Free Radic. Res..

[b0260] Rijken M., Wit J.M., Le Cessie S., Veen S., Leiden Follow-Up Project on Prematurity (2007). The effect of perinatal risk factors on growth in very preterm infants at 2 years of age: the Leiden Follow-Up Project on Prematurity. Early Hum. Dev..

[b0265] Rosell A., Cuadrado E., Ortega-Aznar A., Hernández-Guillamon M., Lo E.H., Montaner J. (2008). MMP-9-positive neutrophil infiltration is associated to blood-brain barrier breakdown and basal lamina type IV collagen degradation during hemorrhagic transformation after human ischemic stroke. Stroke.

[b0270] Selzner N., Selzner M., Odermatt B., Tian Y., Van Rooijen N., Clavien P.-A. (2003). ICAM-1 triggers liver regeneration through leukocyte recruitment and Kupffer cell-dependent release of TNF-alpha/IL-6 in mice. Gastroenterology.

[b0275] Semple B.D., Blomgren K., Gimlin K., Ferriero D.M., Noble-Haeusslein L.J. (2013). Brain development in rodents and humans: identifying benchmarks of maturation and vulnerability to injury across species. Prog. Neurobiol..

[b0280] Septer S., Edwards G., Gunewardena S., Wolfe A., Li H., Daniel J., Apte U. (2012). Yes-associated protein is involved in proliferation and differentiation during postnatal liver development. Am. J. Physiol. Gastrointest. Liver Physiol..

[b0285] Si-Tayeb K., Lemaigre F.P., Duncan S.A. (2010). Organogenesis and development of the liver. Dev. Cell.

[b0290] Sibson N.R., Blamire A.M., Bernades-Silva M., Laurent S., Boutry S., Muller R.N., Styles P., Anthony D.C. (2004). MRI detection of early endothelial activation in brain inflammation. Magn. Reson. Med..

[b0295] Smith C.J., Denes A., Tyrrell P.J., Di Napoli M. (2015). Phase II anti-inflammatory and immune-modulating drugs for acute ischaemic stroke. Expert Opin. Investig. Drugs.

[b0300] Smith C.J., Emsley H.C.A., Gavin C.M., Georgiou R.F., Vail A., Barberan E.M., del Zoppo G.J., Hallenbeck J.M., Rothwell N.J., Hopkins S.J., Tyrrell P.J. (2004). Peak plasma interleukin-6 and other peripheral markers of inflammation in the first week of ischaemic stroke correlate with brain infarct volume, stroke severity and long-term outcome. BMC Neurol..

[b0305] Smith C.J., Emsley H.C.A., Vail A., Georgiou R.F., Rothwell N.J., Tyrrell P.J., Hopkins S.J. (2006). Variability of the systemic acute phase response after ischemic stroke. J. Neurol. Sci..

[b0310] Stolp H.B., Dziegielewska K.M., Ek C.J., Habgood M.D., Lane M.A., Potter A.M., Saunders N.R. (2005). Breakdown of the blood-brain barrier to proteins in white matter of the developing brain following systemic inflammation. Cell Tissue Res..

[b0315] Stolp H.B., Johansson P.A., Habgood M.D., Dziegielewska K.M., Saunders N.R., Ek C.J. (2011). Effects of neonatal systemic inflammation on blood-brain barrier permeability and behaviour in juvenile and adult rats. Cardiovasc. Psychiatry Neurol..

[b0320] Stolp H.B., Turnquist C., Dziegielewska K.M., Saunders N.R., Anthony D.C., Molnár Z. (2011). Reduced ventricular proliferation in the foetal cortex following maternal inflammation in the mouse. Brain.

[b0325] Straley M.E., Van Oeffelen W., Theze S., Sullivan A.M., O’Mahony S.M., Cryan J.F., O’Keeffe G.W. (2017). Distinct alterations in motor & reward seeking behavior are dependent on the gestational age of exposure to LPS-induced maternal immune activation. Brain. Behav. Immun..

[b0330] Trépanier M.O., Hopperton K.E., Mizrahi R., Mechawar N., Bazinet R.P. (2016). Postmortem evidence of cerebral inflammation in schizophrenia: a systematic review. Mol. Psychiatry.

[b0335] Turner R.J., Sharp F.R. (2016). Implications of MMP9 for blood brain barrier disruption and hemorrhagic transformation following ischemic stroke. Front. Cell. Neurosci..

[b0340] van Kasteren S.I., Campbell S.J., Serres S., Anthony D.C., Sibson N.R., Davis B.G. (2009). Glyconanoparticles allow pre-symptomatic in vivo imaging of brain disease. Proc. Natl. Acad. Sci. U.S.A..

[b0345] Vargas D.L., Nascimbene C., Krishnan C., Zimmerman A.W., Pardo C.A. (2005). Neuroglial activation and neuroinflammation in the brain of patients with autism. Ann. Neurol..

[b0350] Vila N., Castillo J., Dávalos A., Chamorro A. (2000). Proinflammatory cytokines and early neurological worsening in ischemic stroke. Stroke.

[b0355] Villapol S. (2016). Consequences of hepatic damage after traumatic brain injury: current outlook and potential therapeutic targets. Neural Regen. Res..

[b0360] Villapol S., Kryndushkin D., Balarezo M.G., Campbell A.M., Saavedra J.M., Shewmaker F.P., Symes A.J. (2015). Hepatic expression of serum amyloid A1 is induced by traumatic brain injury and modulated by telmisartan. Am. J. Pathol..

[b0365] Walthall K., Cappon G.D., Hurtt M.E., Zoetis T. (2005). Postnatal development of the gastrointestinal system: a species comparison. Birth Defects Res. B Dev. Reprod. Toxicol..

[b0370] Wilcockson D.C., Campbell S.J., Anthony D.C., Perry V.H. (2002). The systemic and local acute phase response following acute brain injury. J. Cereb. Blood Flow Metab..

