# Supplementary material for: Hepatic acute phase response protects the brain from focal inflammation during postnatal window of susceptibility
Source: Brain Behav Immun. 2018 Mar;69:486–98. doi: 10.1016/j.bbi.2018.01.008 (PMC5871396; doi:10.1016/j.bbi.2018.01.008)
Supplement: Supplementary data 1 [file mmc1.docx]

**Suppl. Table 1. mRNA expression levels of inflammatory mediators and number of neutrophils is the liver after injection of 100ng IL-1β in the brain at different time points during postnatal period.**

| **mRNA** | **P7** | **P14** | **P21** | **P56** | **P < 0.05** |
| --- | --- | --- | --- | --- | --- |
| IL-6 | 104.40 ± 10.23 | 84.85 ± 5.17 | 8.81 ± 2.21 | 3.65 ± 0.44 | P7 vs. P14, P21 and P56 |
| TNF | 7.86 ± 0.41 | 6.42 ± 0.60 | 2.34 ± 0.09 | 1.11 ± 0.16 | P14 vs. P7, P21 and P56 |
| ICAM-1 | 5.11 ± 0.53 | 3.82 ± 0.21 | 4.23 ± 0.60 | 3.20 ± 0.22 | P7 vs. P14 and P56 |
| CXCL-2 | 82.99 ± 18.00 | 46.22 ± 6.57 | 46.35 ± 19.50 | 3.77 ± 0.77 | P7 vs. P14, P21 and P56 |
|  |  |  |  |  | P14 vs. P7 and P56 |
|  |  |  |  |  | P21 vs. P7 and P56 |
| CXCL-5 | 10.60 ± 1.28 | 12.60 ± 0.85 | 5.41 ± 0.84 | 2.35 ± 0.29 | P7 vs. P21 and P56 |
|  |  |  |  |  | P14 vs. P7, P21 and P56 |
|  |  |  |  |  | P21 vs. P56 |
|  |  |  |  |  |  |
| **Neutrophils** | **P7** | **P14** | **P21** | **P56** | **P < 0.05** |
|  | 362 ± 40 | 338 ± 52 | 237 ± 8 | 155 ± 18 | P7 vs. P21 and P56 P14 vs. P21 and P56 |

Data are represented as relative fold change from expression levels of 8-week old naïve mice for mRNA levels of IL-6, TNF, ICAM-1, CXCL-2 and CXCL-5 and the number of neutrophils is shown as number of cells/mm^2^. Statistical significance was calculated by a two-way ANOVA analysis with Bonferroni–Dunn post-hoc test, where all conditions (naïve, saline, 1ng IL-1β and 100ng IL-1β) and ages were compared. This data shows that the magnitude of the acute phase response 4 hours after intracerebral injection of 100ng IL-1β is higher in younger animals and decreases with age.
